# Structural changes in autism reflect atypical brain network organization and phenotypical heterogeneity: a hybrid deep network approach

**DOI:** 10.1101/2025.11.02.686152

**Published:** 2025-12-03

**Authors:** Siddharth Lokray, Basilis Zikopoulos, Arash Yazdanbakhsh

**Affiliations:** 1 Computational Neuroscience and Vision Laboratory, Department of Psychological and Brain Sciences, Boston University, Boston, MA, 02215 United States; 2 Graduate Program for Neuroscience, Boston University, Boston, MA, United States; 3 Center for Systems Neuroscience, Boston University, Boston, MA, United States; 4 Human Systems Neuroscience Laboratory, Department of Health Sciences, Program in Human Physiology, Boston University, Boston, MA, 02215 United States; 5 Department of Anatomy & Neurobiology, Boston University School of Medicine, Boston, MA, United States

**Keywords:** Autism Spectrum Disorder, Convolutional Neural Networks, Functional Brain Networks, Deep Neural Networks, Saliency maps, Social Responsiveness Scale, structural Magnetic Resonance Imaging

## Abstract

Autism Spectrum Disorder (ASD) is a developmental disorder characterized by heterogeneity in social and emotional responses, language, and behavior. Assessments such as the Social Responsiveness Scale (SRS) can quantify this variability but understanding underlying mechanisms and identifying distinct and shared atypical organization and function of brain networks remains a challenge. Convolutional Neural Networks (CNNs) have been used to analyze imaging data. However, the relationship between structural brain changes observed in structural MRI (sMRI), the affected brain functional networks inferred from these structural changes, and their connection to ASD phenotypes and scores still requires systematic investigation. In this study, we ensembled 3D CNNs with other artificial intelligence (AI) methods to conduct a comprehensive analysis of macrostructural changes in ASD. We found consistently dominant involvement of (a) the left hemisphere, (b) the frontal and temporal lobe, and (c) the default mode, salience, and language networks in ASD. Our findings highlighted brain network similarities and differences between high and low severity ASD and showed that typically developed brains fall at the low-severity end of the high-to-low severity spectrum of ASD. Our systematic AI approach utilized the phenotypic heterogeneity and spectral nature of ASD to uncover significant structural changes across brain regions and functional networks, correlating the structural, functional, and phenotypical heterogeneity of individuals with ASD. This enabled us to identify known and novel global and local brain region and network changes in ASD in relation to phenotypes and clinical scores that can guide diagnostic subtyping.

## Teaser (one sentence summary)

We used machine learning approaches to characterize the phenotypic and imaging-derived structural heterogeneity of ASD with the aim to identify the brain regions and networks linked to its cognitive and behavioral phenotypes.

## Introduction

Autism Spectrum Disorder (ASD) is a developmental disorder that affects social interactions, emotional responses, language, and speech development, leading to behavioral inflexibility in affected individuals (Amaral, Schumann, and Nordahl 2008; Ecker 2012; Lord et al. 2000). Types, onset, and severity of symptoms can vary significantly; thus, ASD affects individuals differently leading to a display of a variety of phenotypical combinations. This heterogeneity makes it challenging to understand the underlying mechanisms of disruption, pinpoint cortical and subcortical regions that comprise atypical brain networks in ASD, and ultimately accurately diagnose and treat affected individuals. Heterogeneity in ASD can be partially expressed as a function of the cognitive, behavioral, and verbal scores in the Autism Diagnostic Interview (ADI) (Ecker 2012; Lord, Rutter, and Le Couteur 1994). A key question is whether ASD heterogeneity indices, based on ADI, can be reliably and consistently reflected in and correlated with the structural and functional changes in brain networks that can be assessed *in vivo* by imaging techniques, such as Magnetic Resonance Imaging (MRI) (Amaral, Andrews, and Nordahl 2024; Duan et al. 2025).

Modern AI techniques, including 2- and 3-Dimensional versions of Convolutional Neural Networks (CNNs) and their derivatives, deep attentional, contrastive, and CNN and Long short-term memory (LSTM) hybrids have been used to analyze imaging data (Aghdam, Sharifi, and Pedram 2019; Aglinskas, Hartshorne, and Anzellotti 2022; Ecker et al. 2010; Khosla et al. 2018; Li et al. 2018; Niu et al. 2020; Supekar et al. 2013; Uddin et al. 2013; Zhao et al. 2018a). Most of these studies focused on accurately classifying ASD against typically developed (TD) individuals, using structural imaging datasets, or identified disruptions in brain network activity predominantly using functional imaging (fMRI). For example, Aglinskas et al. (Aglinskas et al. 2022) developed a neural network that identified ASD- and control-specific neuroanatomical variability and correlated ASD anatomical features with symptoms. Using resting state fMRI data, Zhao et al. (Zhao et al. 2018b) developed a neural network that extracted salient brain regions (Zhao et al. 2018b) that were then mapped to outline brain networks that are disrupted in ASD. However, two key relationships remain poorly explored and understood: a) the presence of structural changes and their correlation across brain areas, as a structural pointer to the functionally affected brain networks, and b) correlations between autism diagnostic scores and brain pathology (Uddin et al. 2013).

To address these gaps, we investigated the relationship between structural changes across regions and ASD ADI scores and their association with brain networks to reveal common and divergent structural and functional changes in individuals with high- and low-severity ASD. Our overarching hypothesis is that sMRI, in fact, contains information that reflects functional network and diagnostic score changes in ASD, which can be deciphered, using state-of-the-art AI, to identify salient brain regions and their networks underlying ASD heterogeneity. Our findings suggest that there is indeed partial required information available in macroscopic sMRI that can be used to reliably identify brain regions, connectivity, and networks involved across the heterogeneous spectrum of ASD, and provides a framework for the identification of systematic, correlated neuroanatomical and functional dimensions across ASD.

## Results

In this study, we examined sMRI datasets (ABIDE II, Table S1) from individuals with different ages and different ASD severity scores to capture ASD heterogeneity. Our approach involved development and optimization of CNNs that can initially evaluate imaging data, through individual brain sections (2D). This approach then evolved into direct evaluation of entire brain volumes by custom-building a 3D CNN for feature extraction from the 3D MRI volumes. These extracted features were subsequently clustered by k-Nearest Neighbors algorithm (kNN), resulting in a 3D-CNN-kNN ensemble that accurately distinguished low and high severity ASD individuals based on their structural MRIs. We then extracted brain regions salient for ASD heterogeneity utilizing our hybrid 3D-CNN-kNN ensemble and projected the extracted saliency measures on cortical parcellations using the Destrieux atlas (Destrieux et al. 2010). This enabled a more detailed identification of brain regions with macro-structural changes as highlighted by AI saliency maps, along with the analysis of correlations between these changes, and the severity of ASD. We then identified hierarchical relationships between brain regions, based on our correlation analysis, to associate correlated regions with functional brain networks. We focused, in particular on networks that are affected in ASD, including the default mode network (DMN), salience network (SN), social network, and central executive network (CEN or frontoparietal network), which play key roles in self-awareness, attention, social interactions, and cognitive control, which are all impacted in ASD. We also included the Language, Sensorimotor, Dorsal/Ventral Attention, and Limbic/Emotion networks—known to be affected in ASD—in the same analysis.

**Figure 1:**
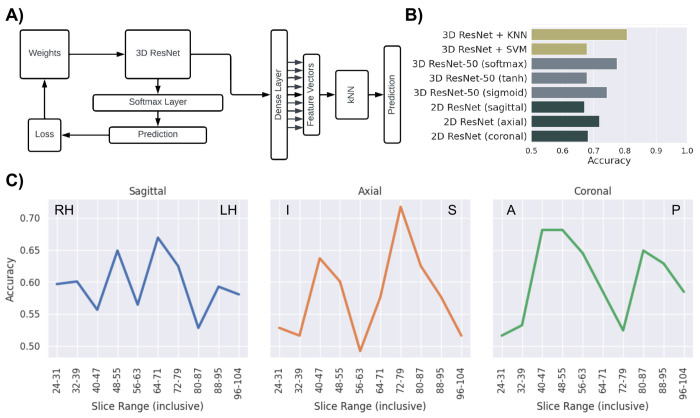
Machine learning network architecture and performance comparison after training. A) 3D ResNet-50 + kNN hybrid architecture. The 3D ResNet-50 was trained initially on the preprocessed structural MRI data, then was passed through a kNN to make the category predictions. B) Performance of 3D ResNet-50 (softmax, tanh, and sigmoid activation), hybrid 3D ResNet-50 (kNN and SVM), and 2D ResNet-50. The performance of the 2D ResNet-50 is representative of the highest performance on the slice ranges. C) Performance of 2D ResNet-50 on the sagittal (slice 24 is the furthest slice on the right hemisphere and slice 103 is furthest slice on the left hemisphere), axial (slice 24 is the most inferior slice and slice 103 is the most superior slice), and coronal (slice 24 is the most anterior slice and slice 103 is the most posterior slice) axes for each slice range. Table S2 provides the accuracy values for each slice range on the sagittal, axial, and coronal axis.

We trained a 2D ResNet-50, our custom developed 3D ResNet-50, and hybrids with kNN and Support Vector Machine (SVM; Fig. 1A), using T1-weighted sMRI scans from individuals with low and high severity ASD (Fig. S5) and compared their performance (Fig. 1B). The 2D ResNet-50 was trained separately on the sagittal, axial, and coronal axes, using MRI slices as input, whereas the 3D ResNet-50 and its hybrid variations were comprised of 3D convolution filters and pooling layers, thus, used MRI *volumes* as their inputs rather than MRI slices. After extensive hyperparameter searches, we noted the performance of the 3D network was superior to the 2D networks; hence, extracted saliency maps from the 3D ResNet-50 and overlaid the salient areas in cortical and subcortical regions a) on the FreeSurfer pial surface, and b) in subcortical volumes to parse brain regions with saliency. We ran correlation analysis between the Social Responsiveness Scale (SRS) Total and SRS subscores and salient regions as well as pairwise cross-region saliency correlation analysis to identify the regions and networks correlated with ASD severity at a macrostructural level.

### Performance of the 2D ResNet-50 on MRI slice intervals.

The performance of the 2D ResNet-50 on each group of slice ranges is shown in Fig. 1B–C. With regard to the performance of the 2D ResNet-50 on the groups of sagittal slices (Fig. S1, Table S2), there were two distinct peaks; one slightly left of medial region of the right hemisphere (slices 64-71) around the thalamus and corpus callosum, and one in the middle of the right hemisphere around the hippocampus, putamen, and amygdala (slices 48-55). On the axial slices (Fig. S2, Table S2), there were two peaks; one around the inferior region (slices 40-47) intersecting the posterior lobe of the cerebellum and a more pronounced peak around the superior region (slices 72-79) intersecting the lateral ventricles and the body of the corpus callosum. The coronal slice groups (Fig. S3, Table S2) showed a solid peak around the anterior region (slices 40-55) crossing the middle frontal gyri, inferior frontal gyri, and the head of the caudate nucleus as well as another peak around the cerebellar region (slices 80-87) crossing the hippocampus, thalamus, and fourth ventricle. The performance of the 2D ResNet-50 on groups of MRI slices provided a basic understanding on what regions of the brain were more useful to differentiate high severity subjects from low severity subjects.

### Performance of the 3D ResNet-50 and hybrid variations on MRI volumes.

The architecture for the 3D ResNet-50 is shown in Fig. 1A. Three activation functions were tested to push the 3D ResNet-50 to perform to its full potential: softmax, tanh, and sigmoid. Softmax, sigmoid, and tanh performed with an accuracy of 77.41%, 74.19%, and 67.74, respectively. Based on these results, we selected the softmax function as the classification layer activation for the retrieval of saliency maps for each subject. Additionally, the two hybrid neural networks tested were the 3D ResNet-50 + kNN and the 3D ResNet-50 + SVM. The difference between the 3D ResNet-50 and the hybrid 3D ResNet-50 is that the classification layer of the neural network is replaced with a fully connected layer with *n* nodes to output a feature vector into the respective machine learning algorithm (kNN or SVM) of the hybrid network (Fig. 1A). The performance with the kNN is significantly better than with the SVM, as the kNN yielded an accuracy of 80.64% (3.23% better than 3D ResNet-50 only) while the SVM yielded an accuracy of 67.74%. The fully connected layer that outputs the feature vectors was initialized with randomized weights thus, many attempts were conducted, and the best accuracy was reported. We used the 3D ResNet-50 for the following saliency, cross-region and region-score correlation analyses.

### Saliency in cortical and subcortical regions.

**Figure 2:**
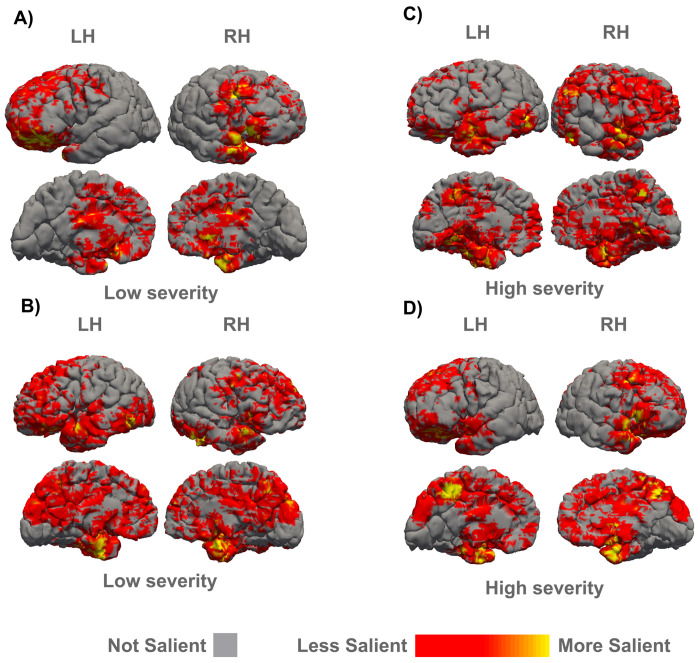
Saliency maps for network classification. Each case saliency map (averaged on sagittal plane) generated from the 3D ResNet-50 was overlaid on the case pial surfaces (gray), which was generated through Freesurfer’s reconall pipeline. Lateral (top row) and medial (bottom row) views of subject’s left hemisphere (LH) and right hemisphere (RH) were visualized by FreeView, Freesurfer’s visualization software. Yellow marked areas highlight more salient regions whereas red marked areas display moderately salient regions. A) Low severity case with SRS-Total Score of 44. B) Low severity case with SRS-Total Score of 42. C) High severity case with SRS-Total Score of 83. D) High severity case with SRS-Total Score of 87.

Sixteen saliency maps were obtained by TensorFlow gradient tape within the 3D ResNet-50 and were overlaid on their respective pial surface (Fig. 2). Salient regions were observed in the dorsolateral prefrontal cortex (BA areas 8, 9, and 46) as well as the orbitofrontal cortex (BA 11, and 13). There are also significant salient regions in the temporal lobe (BA 28, 35, and 36) including the amygdala. Both Broca’s Area (BA 44, and 45) and Wernicke’s Area (BA 22) show regions with moderate saliency. The dorsal cingulate (BA 24, and 32), subgenual cingulate (BA 25), and corpus callosum had distinguishable, moderately salient regions, that were well delineated. Detailed saliency maps overlaid over the MRI scans are shown in Figures S1-3.

**Figure 3:**
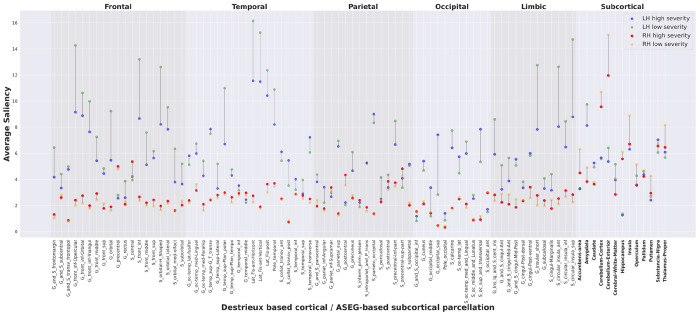
Differences in average saliency between high severity and low severity subjects in the left and right hemispheres for each cortical region labeled based on parcellation by Destrieux atlas and by FreeSurfer’s automatic subcortical segmentation of a brain volume (aseg atlas, bolded regions). Average saliency in high severity subjects in the left hemisphere (LH, blue) are connected to the average saliency in low severity subjects in the left hemisphere (green) and average saliency in high severity in the right hemisphere (RH, red) is connected to the low average saliency in lower severity subjects in the right hemisphere (yellow). In summary, cold colors represent the LH and the hot colors represent the RH and high/low saturation colors represent high/low severity. Note an overall larger average and broader range of saliency in the left hemisphere. The high and low severity values of the brain stem and fornix are 4.72 and 4.79, and 3.34 and 3.55 respectively. These regions are not included in this figure as they do not have a hemisphere attribute.

We used the Destrieux atlas (Destrieux et al. 2010) to create masks of cortical and subcortical regions for 150 subjects (Destrieux et al. 2010) (75 cortical regions per hemisphere) and Freesurfer’s automatic subcortical segmentation (ASEG, 13 subcortical regions per hemisphere as well as brain stem and fornix that were not separated across hemispheres). We calculated the average saliency value per region across all subjects (Fig. 3). Overall, the left hemisphere generally had a) higher saliency value, with an average saliency of 5.85, while the average saliency of the right hemisphere was 2.31 (*p* = 5.19e-17 , df=148), and b) broader saliency range that was estimated by the average saliency range of each region in the left and right hemispheres across high and low severity subjects (*p* = 7.53e-12, df=148). In the left hemisphere, the opercular, orbital, and triangular regions of the inferior frontal gyrus, the horizontal, vertical, and posterior ramus of the lateral sulcus, the anterior and superior segments of the circular sulcus of the insula, and the short insular gyri, temporal pole, and orbital sulci were among the regions with high/moderate saliency. High saliency regions in the right hemisphere included the cerebellum, basal ganglia, thalamus, insula, postcentral and precentral gyrus, the central sulcus, and the temporal pole.

### Correlation between corticocortical saliency and corticosubcortical saliency.

**Figure 4:**
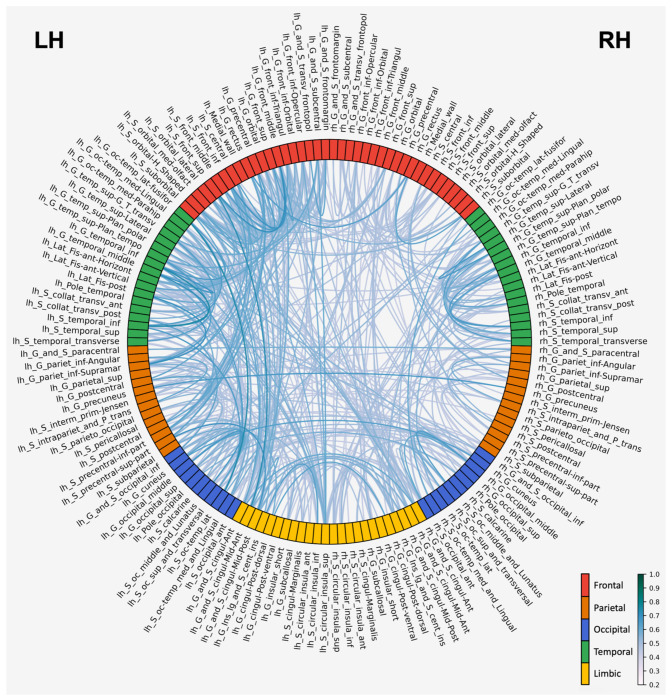
Circular connectivity plot highlighting significant pairwise saliency correlations (critical α value = 0.01) between cortical brain regions across high and low severity MRI cases. Darker lines represent higher Kendall τ whereas lighter lines represent lower Kendall τ values. The color of each brain region corresponds to the lobe the region is located in. The strongest correlations were mostly between regions within the same lobe however, cingulate regions showed several strong cross-lobe correlations.

**Figure 5:**
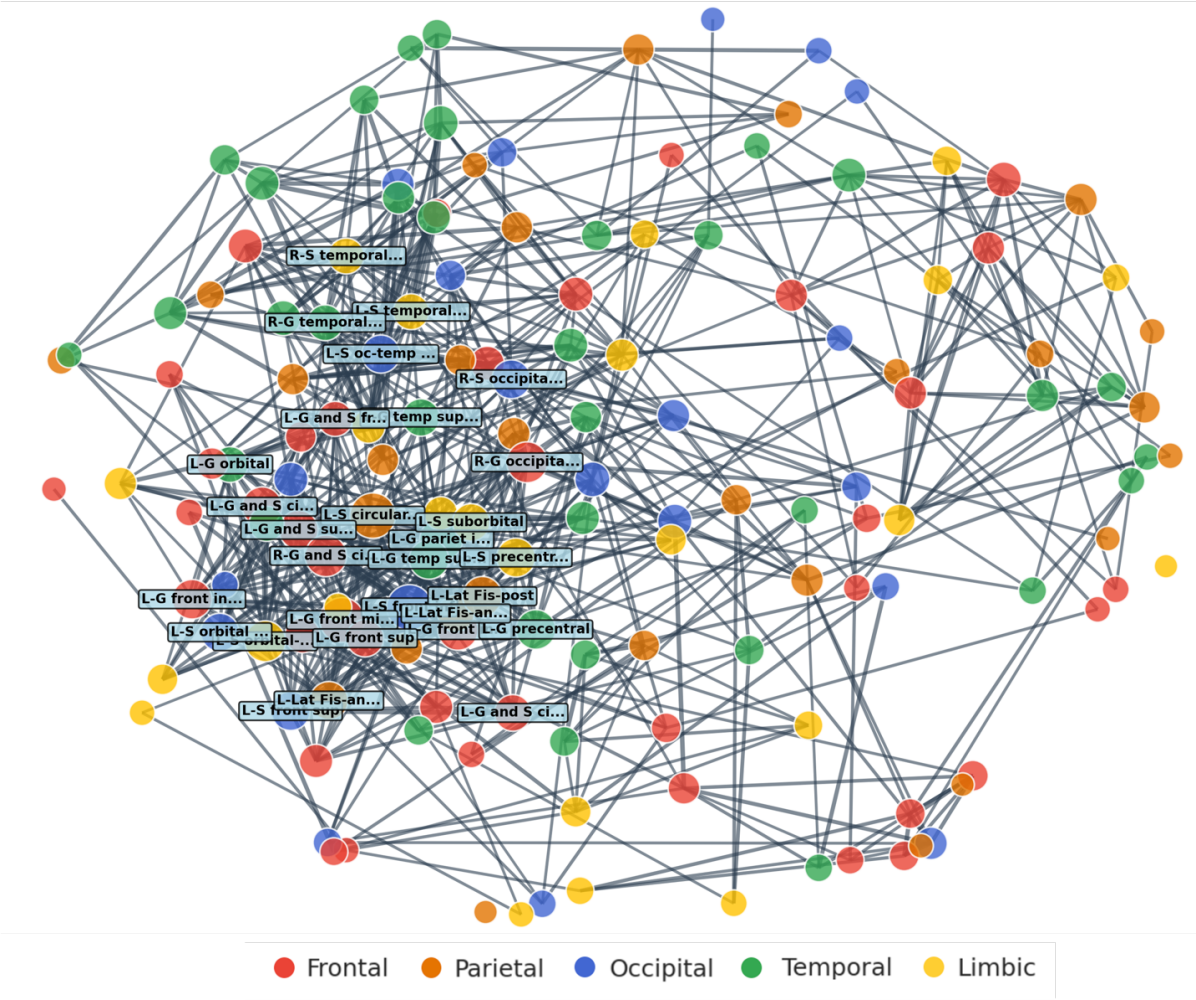
Spring Cluster layout graph for corticocortical saliency correlations. Connectivity brain regional analysis dependent on region-region Tau correlations depicted in Figure 4. Each node represents a region in the Destruiex atlas, highlighted with their corresponding lobe color. Size of each node is dependent on how many significant correlations (α < 0.01 and where τ > 0.4) the respective region takes part in. The distance between correlated regions is weighted by 1– τ, where highly correlated regions are closer together. The algorithm arranges the nodes to minimize the weights between the regions. This leads to clustering of highly correlated regions (top 30 regions are named) towards the center and less correlated regions to the periphery.

Kendall τ rank-based correlation between cortical regions in the Destrieux atlas were conducted to observe the relationship in paired saliencies within the brain. The top 500 significant correlations sorted by the τ value are shown in Fig. 4. Each connecting thread indicates the presence of significant (*p* < 0.01) correlation with τ > 0.4 as a midlevel threshold. To maximize and optimize inference from these complex connectivity maps, based on regional saliency correlations, we took the following steps. First, we used a stepwise adaptive threshold approach (from 0 to 0.6 with steps of 0.1) for examination of cross-regional correlations τ. We found that there were only few cross-regional correlations beyond threshold 0.6. The reason for the extensive, stepwise examination of τ thresholds, is because there were salient regions that were significantly correlated with *many* other regions but with low τ, and also regions correlated with a *few* other regions but with a higher value of τ. To distinguish between the two groups, we refer to the first as the regions *correlated with many* and the second, as the *highly correlated* regions groups, respectively. Both groups reflect extreme and in between scenarios that offer insights about the affected brain networks in ASD. We generated Spring Cluster layout graphs (Fruchterman and Reingold 1991; Hamaguchi, Marumo, and Takeda 2025; NetworkX Developers 2025), which arranged cortical regions based on the number of significant cross-regional Kendall Tau correlations that each region maintained with other regions (Fig. 5). The algorithm created a network where each cortical region was a node, and significant correlations above the threshold became binary connections between regions. Regions were positioned such that those with a greater number of significant correlations with other regions were pulled toward the network center due to having more attraction forces acting upon them. This created a natural hierarchy, where regions serving as potential network hubs appeared in the center while regions with a smaller number of significant correlations appeared at the periphery. This spatial organization allowed identification of cortical regions that may function as structural pathology hubs in ASD, based on their tendency to show significant correlations with many other brain regions rather than the magnitude of individual correlation values. Fig. 5 shows the Spring Cluster layout with τ threshold of 0.4, approximately midway within 0.1-0.6. At this threshold, the following regions were placed more toward the center, because they had a larger number of significant correlations with other regions: left inferior frontal sulcus, left superior segment of the circular sulcus of the insula, left middle frontal gyrus, left supramarginal gyrus, right anterior part of the cingulate gyrus and sulcus (ACC), right middle occipital gyrus, left orbital sulci, left opercular part of the inferior frontal gyrus, left posterior ramus of the lateral sulcus, left temporal plane of the superior temporal gyrus. For the full range of thresholds (0.1-0.6) to cover both groups of “correlated with many” and “highly correlated regions”, see supplementary table S3.

**Figure 6:**
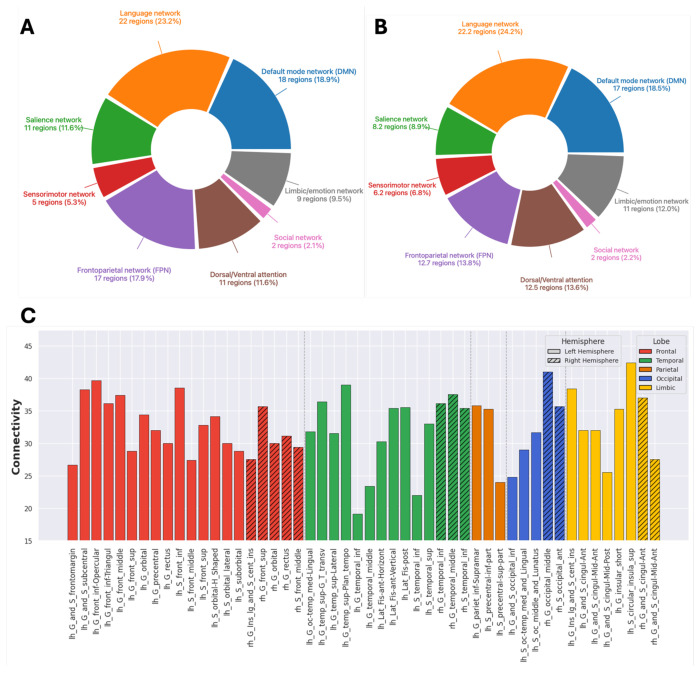
Degrees of involvement of functional brain networks and lobes in ASD. **A.** The degree of involvement for each brain network was derived from the pairwise brain region saliency correlation analysis shown in Fig. 4. Specifically, we considered significant correlations (*p* < 0.01) with τ exceeding a set threshold (e.g., 0.4) and selected the top *n* brain regions (e.g., 50) with the highest number of such correlations. From this list, we counted how many regions belonged to each functional network (raw counts shown beside the pie chart) and calculated the proportion of each network’s count relative to the total, expressed as a percentage. **B.** The analysis in **A** was repeated and averaged across thresholds ranging from 0.1 to 0.6 in increments of 0.1. These measures indicate the global engagement of regions within functional networks with the rest of the brain in ASD. **C.** The number of connections/correlations per brain region was summed across the top 50 areas from each threshold-based table in S3, then divided by six (the number of τ thresholds between 0.1 and 0.6) to calculate the average number of correlations/connections per region across all thresholds. Areas were subsequently re-ranked based on these averaged values to identify the top 50 regions involved in ASD according to averaged structural correlations. The largest number of regions involved in ASD are found in the frontal and temporal lobes in the left hemisphere.

To better understand the organization and likely functional effects of salient regions that showed a high number of correlations with other regions or high correlation values in ASD, we counted the number of salient regions belonging to 4 main brain networks, consistently affected in ASD, i.e., Default Mode (DMN), Salience (SN), Frontoparietal (FPN, or central executive network), and Social networks, as well as 4 additional networks, i.e., Language, Sensorimotor, Dorsal/Ventral attention, and Limbic/Emotion networks, which have been associated with ASD dysfunction. For example, using τ = 0.4, Fig 6A pie chart shows the relative involvement of the 8 networks, highlighting the relative representation of regions that are part of each of these 8 networks, which are well-established in ASD pathology. The raw counts, such as 22 for the Language network (LN) is the count of brain regions belonging to LN in S3 table with threshold τ = 0.4, and the ratio 22/95 = 23.2% indicates the ratio of LN involvement in which 95 is the total brain region count across all 8 networks in the pie chart.

For the overall examination of brain network involvement across the full range of τ thresholds (0.1-0.6), we averaged the brain region counts and their ratios per network across the 6 pie charts, similar to the one in Fig 6A, for the 6 thresholds, 0.1-0.6 (Fig. 6B). Overall, the Language and Default Mode Networks consistently showed the highest average representation and association with ASD, among functional networks, followed by the Frontoparietal, Dorsal/Ventral attention, Limbic/emotion, and Salience, networks. The Sensorimotor and Social networks were minimally represented, reflecting their more distributed or overlapping nature in the anatomical brain regions.

Additionally, we summed the number of connections/correlations per brain region across the top 50 areas from each threshold-based table in S3, and divided by 6 (number of τ thresholds between 0.1-0.6) to reflect the average number of correlations/connections per brain region across tables covering all thresholds. We then, re-ranked the areas based on these averaged correlation/connection values to identify the top 50 regions involved in ASD based on averaged structural correlations (Fig. 6C). This analysis showed that pairwise saliency correlation across cases was dominant in the left hemisphere, both in terms of number of inferred structural connections per region (higher values in the average connection columns) and in terms of the number of regions as central hubs (more table rows for left than right). About 40% of the correlations were between regions in the same lobe (per hemisphere), with the left frontal lobe exhibiting the highest number of interregional correlations (12.6%).

**Figure 7:**
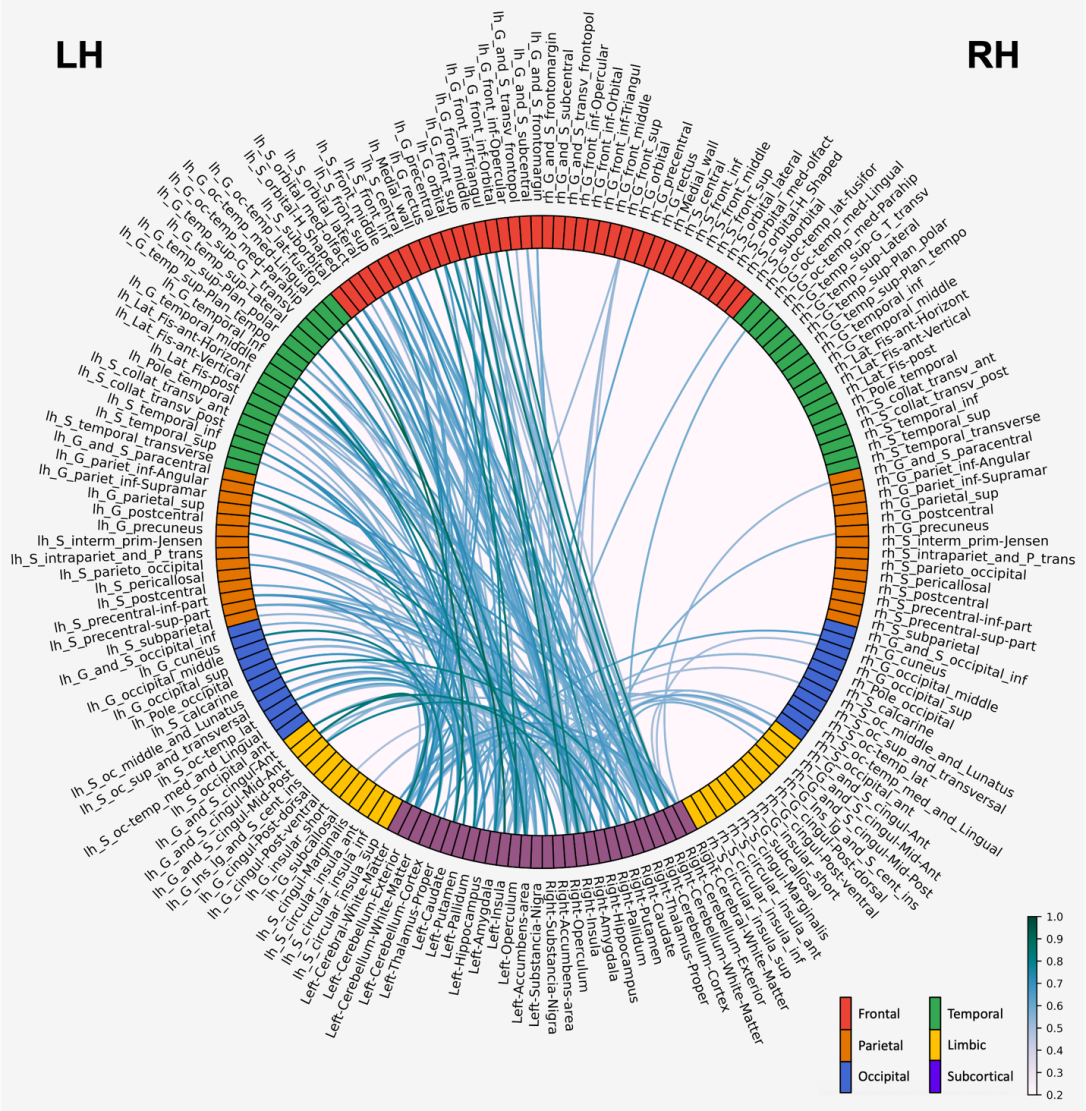
Significant pairwise saliency correlations (critical α value = 0.01) between cortical brain regions and subcortical brain regions across high and low severity MRI cases. This figure is organized like Fig. 4, with darker lines representing higher Kendall τ and lighter lines representing lower Kendall τ values. Color of each brain region corresponds to lobe the region is located in. Notably, the corticosubcortical correlations have higher τ values than the corticocortical correlations, shown in Fig. 4.

Fig. 7 visualizes the Tau correlations between the cortical and subcortical regions. The strongest and most numerous correlations of the subcortical regions were with the cortical regions in the left hemisphere [true for all lobes except for limbic, which had balanced left-right correlation with subcortical regions]. 77.27% of the subcortical to cortical correlations were connected to regions in the left hemisphere while 9.09% of the correlations were connected to regions in the right hemisphere, with the rest being within the subcortical regions.

### Correlations regarding regional saliency averages.

**Figure 8.**
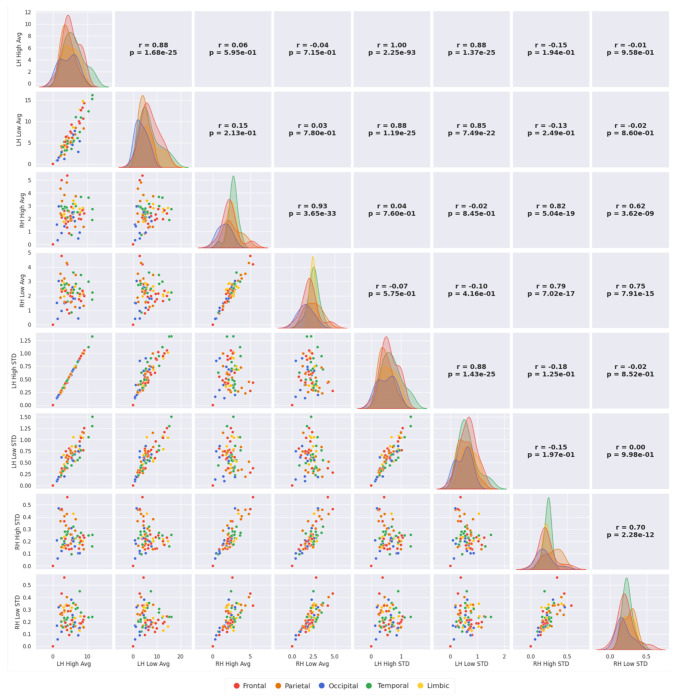
Scatter plots and distributions of the average regional saliency and the associated standard deviation for each cortical brain region. Plots are organized on an 8×8 grid, based on specifications in Fig 3. Each point in the scatter plot belongs to a brain region from Destrieux atlas similar to Fig. 3, total of 75 regions. Scatter plot axes are restricted to the left (LH) or right (RH) hemisphere, high (High) or low (Low) severity score cases, and the metrics include average (Avg) and standard deviation (STD) across the targeted cases (high or low severity). For example, an axis label of LH High STD refers to regions in the left hemisphere of all high severity cases averaged for each brain region. Therefore, each scatter plot has 75 points corresponding to one of the 75 brain regions listed in Fig. 3. Brain regions belonging to each lobe are color coded in all scatter and distribution (diagonal of the grid) plots. Key findings include a) maximum trend of *linear* scatter within LH high score, less in LH low score, RH high and low scores, and isotropic or no trend scatter across LH and RH (either with high or low score), b) distribution plots on the grid diagonal are more normal like for RH and low severity and max deviated from normal for LH high severity. For interpretations, see the text.

We investigated correlations between average and standard deviation of saliency within either LH or RH regions from cases either with high or low severity, resulting in an 8×8 grid accounting for LH vs RH (2) × Average (Avg) vs Standard deviation (STD) (2) × High vs Low severity (2) scatter plots and distribution histograms (Fig. 8). Overall, the patterns of distribution and scatter indicate more proportional area saliency average and standard deviation in LH in high severity cases, less proportionality in RH in high severity, and no proportionality of area saliency average in one hemisphere and standard deviation of equivalent area in opposite hemisphere in high and low severity cases. This comparison showed that LH structural modifications in high severity cases generate STD proportional to Avg saliency across subjects, indicative of proportional modifications across regions, such as inflated or compressed saliency. The degree of proportionality was smaller in low severity LH and high and low severity RH, and nonexistent across LH and RH. Together, these findings revealed systematic heterogeneity, especially pronounced in LH in high severity ASD and indicate that the heterogeneous structural impact of ASD on each left-brain region was proportional to the mean structural impact on that left brain region across affected individuals. In this regard, the structural saliency of all regions within each lobe in the LH for high and low severity were not normally distributed (see diagonal panels with distribution plots in Fig. 8), especially compared to the right hemisphere. This strongly suggests that structural aberrations in LH regions were not an artifact of random distribution across MRI scans and there were macro-structural effects reflected in MRI saliency. This is consistent with the dominant involvement of left hemisphere in ASD.

### Correlating regional saliency and ASD severity across cases.

We analyzed correlations using Kendall rank correlation coefficient τ between the SRS subscores (cognition, awareness, communication, mannerisms, and motivation) and the saliency of each brain region across low to high severity ASD cases. For these analyses, we considered 150 regions (2×75 for both left and right hemispheres) labeled using the Destrieux atlas parcellation and found that cognition, awareness, communication, mannerisms, and motivation subscores were significantly (*p* < 0.05) correlated with a distinct number of salient regions in each lobe of the left and right hemisphere (8/7, 12/0, 8/4, 22/7, 7/2, respectively; Table 1).

In summary, comparison across lobes revealed saliency correlation of more regions of the frontal lobe with SRS subscores, both in left and right hemispheres, as well as the dominance of the left hemisphere, in terms of the number of regions that had significant correlations with SRS subscores.

### Typically developed vs. low severity brains.

We used the hybrid 3D-CNN we developed and trained to classify sMRI scans only from individuals with high- and low-severity ASD to now classify scans from control, neurotypical subjects (therefore with no SRS score). Scans from this control group were predicted with 86.47% accuracy as low severity subjects by the 3D ResNet50 trained on low and high severity ASD subjects. Saliency values of all regions mapped according to the Destrieux atlas for the low severity vs. control subjects showed a linear relationship, suggesting that the two groups share similar defining characteristics and are easily distinguishable from the high severity group (Supp. Fig. S4). This high resemblance between the control group and low severity allowed us to effectively treat the structural aberrations in ASD along with typical structural variations in neurotypical brain as a spectrum.

## Discussion

Advances in genetic, molecular, histopathological, and imaging studies, as well as clinical diagnosis of neurodevelopmental, neurodegenerative, and mental disorders, provide a wealth of data that can facilitate fine quantitative characterization of their heterogenous nature. Combined with major developments in the field of explainable AI, through techniques that can highlight the salient input features contributing to classification, make it possible to systematically interrogate and identify the underlying structural and functional correlates of such heterogeneities

Within this framework, in this study we developed explainable AI approaches and applied these to study *in vivo* structural MRI sequences from individuals with ASD from the ABIDE II dataset with the goal to correlate key structural changes in the autistic brain with the functional, cognitive, and behavioral heterogeneity of ASD. We parsed heterogeneity through a) correlations between the phenotype spectrum, in terms of diagnostic subscores, with the AI derived spectrum of structural and anatomical saliency, and b) pairwise correlations between the AI derived spectrum of structural saliency as a pointer to connection and brain network changes in ASD. Using this approach, we developed and utilized state-of-the-art AI networks, algorithms, and their ensembles to find an optimal combination that achieved the highest accuracy in low vs. high severity ASD classification. Then, based on the best AI ensemble, we extracted salient brain regions that contributed to the classification and measured a) the correlation of their saliency level with the cognitive and behavioral heterogeneity, and b) their pairwise saliency correlation as pointers to brain functional networks and their heterogenous modifications in ASD. After extensive testing of 2D and 3D CNNs and AI algorithms, we found that the 3D ResNet-50, as a feature extractor for the structural MRI, and the kNN algorithm, as a classifier of the features, formed the optimal ensemble that yielded the highest accuracy for classifying ASD severity from structural MRI. Then based on this optimal ensembled AI, we extracted the regions of interest, mapped through cortical and subcortical segmentation, by generating MRI saliency maps using a CNN gradient method.

Our findings about brain structural changes in ASD can be summarized as a) differential involvement of left vs right hemispheres with more involvement of left hemispheric regions and networks, b) more extent (connection number/correlation count) in the ASD involvement of the left brain, c) more left brain structural changes correlated with ASD subscores and phenotype, and therefore d) more reflection of ASD heterogeneity in left brain correlated with phenotypical and structural changes. Among brain lobes and networks, the frontal and temporal lobes and the language, default mode, central executive, and dorsal/ventral attention, limbic emotion, and salience networks showed most structural changes in ASD. Our systematic Al approach leveraged the phenotypical heterogeneity and spectral nature of ASD to uncover structural brain region heterogeneity in ASD, as well as correlations between areas and across structural and phenotypical/diagnostic heterogeneity. This revealed global, regional, and network changes in ASD associated with symptom severity and diagnostic scores. Finally, our structural analysis showed that the neurotypical brain fell on the low-end tail of the low-high severity ASD spectrum.

The constellation and configuration of high vs. low saliency and correlations we found, revealed a large number of networks and areas that are affected in ASD, including but not limited to multiple brain regions that are posterior components of the default mode network, bilateral precuneus, right inferior occipital gyrus, left cerebellar regions (Liloia et al. 2023). Significant positive or negative structural saliency correlations revealed in this study can be in part the origin or outcome of functional connectivity changes (over- or under-connectivity) in ASD, together motivating the consideration of brain structural phenotypes of ASD and laying a framework for neuroanatomical heterogeneity in ASD (Shan et al. 2022). Overall, ASD exhibited heterogenous and widespread structural and functional feature prints. The saliency overlay results, showed distribution across areas and hemispheres with high saliency in frontal, limbic and temporal areas. Areas with a role in speech, language, comprehension, and non-verbal communication displayed high saliency. These included pars opercularis, pars orbitalis, pars triangularis, inferior frontal regions (including Broca’s area), superior temporal gyrus (Bigler et al. 2007; Foundas et al. 1996; Peeva et al. 2013; Yamasaki et al. 2010), and the horizontal, vertical, and posterior rami of the lateral sulcus, which define the pars triangularis, and pars opercularis regions and also hold and Wernicke’s area (Chaichana and Quiñones-Hinojosa 2019; Griffiths et al. 2010). Other areas with high saliency included the insula that has a role in sensory integration and regulation of emotion (Viñas-Guasch and Wu 2017), and the temporal pole, which is commonly associated with visual processing, socioemotional processing, and other cognitive processes (Herlin, Navarro, and Dupont 2021). Interestingly, left hemisphere regions throughout most of the cortex showed higher saliency values and larger separation across the spectrum of low-high ASD severity whereas regional saliency in the right hemisphere remained lower and with smaller separation across the ASD spectrum.

The saliency-based correlations highlighted involved networks and connectivities and facilitated ranking of regions in terms of affected connectivity. In line with prior reports [reviewed in (Amaral et al. 2008)], our findings also showed the heterogeneous and distributed neuroanatomy of ASD (Amaral et al. 2008), which is associated with functional connectivity and network aberrations (Müller 2007), including widespread reported over- and under-connectivity (Baran et al. 2023; Cherkassky et al. 2006; Just et al. 2007, 2007; Kenet et al. 2012; Khan et al. 2013, 2013; Müller 2007, 2007). Our analysis revealed functional networks implicated in ASD, with the language, default mode (DMN), central executive (CEN or frontoparietal), dorsal/ventral attention, limbic/emotion, and salience (SN), networks at the top of the list. These findings are in line with previous studies by Uddin et al. (2013) that also highlighted the involvement of these and other functional networks, connections, and regions in ASD. Further support for our findings comes from observations of weaker resting state connectivity across distributed networks in ASD, including frontoamygdala functional connectivity that likely contributes to socioemotional impairments (Odriozola et al. 2019). Resting state underconnectivity has been reported in functional studies using the ABIDE dataset (Duan et al. 2017), indicating underconnectivity in areas within the default mode network (DMN) and within the ventral attention network (VAN), as well as between pairs of networks, including DMN and Visual Network (VN), dorsal attention network (DAN) and VAN, and limbic network (LN) and frontoparietal network (FPN). Schipul et al. (Schipul et al. 2012) showed decreased functional and structural connectivity measures consistent with the corpus collosum size change in ASD compared to TD, which could account for some of the hemispheric differences we observed. Functional and anatomical underconnectivity has also been noted by (Just et al. 2007), using functional measures (synchronization) between parietal and frontal areas in proportion with the smaller size of the genu of corpus callosum. Local and long-range functional connectivity was shown to be reduced within FFA, and between the FFA and three distant cortical regions, the left precuneus, left inferior frontal gyrus (IFG), and left anterior cingulate cortex (Khan et al. 2013), which were also salient in our analysis. The anterior cingulate cortex, in particular, has been a consistent site of ASD pathology in imaging (Thakkar et al. 2008) and histopathological and computational studies (Yazdanbakhsh et al. 2024; Zikopoulos, Liu, et al. 2018; Zikopoulos and Barbas 2010; Zikopoulos, García-Cabezas, and Barbas 2018). Moreover, several resting-state networks may be more loosely connected in ASD, based on lower connectivity of the anterior cingulate region with posterior cingulate and precuneus areas, between anterior and posterior cingulate, and lower functional connectivity between anterior and posterior medial cortices (Cherkassky et al. 2006). Following the same underconnectivity trend, reduced functional connectivity between the frontal eye fields (FEF) and dorsal anterior cingulate cortex, regions critical to volitional ocular motor control, has been reported in ASD (Kenet et al. 2012). In addition, a study that used whole sMRI and SVM for pattern classification, in a similar manner to this work, found distributed networks, limbic, frontal-striatal, fronto-temporal, fronto-parietal and cerebellar systems that distinguished ASD from controls, with better gray matter- compared to white matter-based classification than white matter only (Ecker et al. 2010). On the other hand, regions and networks that were salient in our analyses have also been implicated in reports of hyperconnectivity in the salience network (SN), composed of anterior cingulate cortex ACC, ventral anterior insular cortex, amygdala, hypothalamus, ventral striatum, thalamus, and brainstem nuclei (Uddin et al. 2013), as well as social network-related long- and short- range hyperconnectivity (Supekar et al. 2013).

### Methodological considerations and limitations

In this study we used both 3D and 2D neural networks. The 3D ResNet-50 allowed for the whole MRI volume to be used for training whereas 2D neural networks only trained on slices of the MRI. The volumetric nature of the 3D ResNet-50 highlighted patterns that extended across slices, so the 3D ResNet-50 outperformed the 2D ResNet-50 in the sagittal, axial, and coronal planes, demonstrating the importance of data across MRI slices rather than the data on the slice itself. However, 2D neural networks have become very sophisticated in recent years while 3D neural networks are extremely prone to variability in data. It was therefore essential, for accurate and consistent performance of the 3D neural network, to reduce variability through MRI preprocessing.

In order to use the 3D ResNet-50 as a feature extractor, we modified the model to output feature vectors to a SVM and a kNN. The SVM and kNN essentially compared each test MRI subject with the training subjects. The SVM fitted a hyperplane to the training data and made its prediction of the testing data based on the hyperplane while, the kNN compared the testing data on a *n*-dimensional plane to the *k* nearest training data (Euclidian distance). With a relatively low number of subjects available, the SVM tended to overfit the hyperplane to the training data causing inaccurate predictions of the testing data. However, the kNN was successful in making correct predictions as it merely compared and not fitted the testing to the training data.

Importantly, our study focused on male subjects, due to limited availability of MRI sequences and accompanying diagnostic/phenotypic data for females therefore, reflecting heterogeneity also seen in other studies of male individuals (Chen et al. 2019). Moreover, the amount of publicly available MRI datasets that include phenotypic data such as the SRS Total and its subscores is limited, which naturally could make our ensembled AI prone to overfitting, impacting extracted regions of interest, based on saliency maps and saliency magnitude.

Additionally, the saliency maps depict the contribution of each voxel to making the low to high severity ASD prediction, as a macrostructural pointer to ASD, and the highly significant saliency correlations across pairs of regions can only suggest their involvement in ASD as part of brain functional networks. Nuances such as intra-areal and inter-areal hypo- and hyper-connectivity changes in ASD (Cherkassky et al. 2006; Just et al. 2007; Kenet et al. 2012; Khan et al. 2013; Müller 2007; Supekar et al. 2013) appear as a tip of iceberg in saliency correlation analysis, and neuronal, connectional and neurobiological changes in ASD demands approaches with much higher spatial resolution. Therefore, the current study can at best outline underlying brain region and network heterogeneity in the severity of ASD.

Finally, it is worth noting that we did not train the network on any typically developed (TD) brain MRIs. Instead, we used TD brain MRIs as a test input into our final, developed 3D network ensemble and extracted brain region saliency to compare with ASD population results. This combination revealed the congruency of saliency assignment by the network for TD and low severity ASD subjects, with TD saliency on the low-end tail of the distribution. This key finding suggests a continuous and spectral heterogeneity of structural brain changes across subjects, with low severity ASD and TD individuals lying at one end of the structural spectrum.

## Conclusions

Our findings showed that structural MRI contains information that reflects functional network and diagnostic score changes in ASD, which can be identified, using state-of-the-art AI, to pinpoint salient brain regions and their networks underlying ASD heterogeneity. Our analyses provided distinct involvement measures of frontal, temporal, parietal, and occipital lobes, and limbic, insular, and subcortical regions and their constituent areas and subregions in social-awareness, attention, social interactions, and cognitive control scores. Additional correlation and subsequent ranking revealed hierarchically arranged involvement of functional networks in ASD, which, in descending order of impact, included language, default mode (DMN), central executive (CEN or frontoparietal) & dorsal/ventral attention, limbic/emotion networks, salience (SN), sensorimotor, and social, networks. These findings highlight novel, detailed, and systematic anatomical, functional, and phenotypical changes and associations that can be used as biomarkers to parse and frame the heterogeneity and spectral nature of ASD.

## Figures and Tables

**Table 1.** Brain regions associated with each ASD sub-score, grouped by hemisphere and lobe.

Sub-score	Left Hemisphere	Right Hemisphere
Cognition	**Frontal**: Suborbital sulcus, Superior frontal gyrus, Frontomarginal sulcus and gyrus, Transverse frontopolar gyri and sulci **Temporal**: Transverse temporal sulcus **Parietal:** Angular gyrus, Superior parietal lobule, Paracentral lobule and sulcus	**Frontal**:Superior frontal sulcus, Orbital part of inferior frontal gyrus, Suborbital sulcus, Inferior frontal sulcus **Temporal**: Horizontal ramus of lateral sulcus **Parietal**: Angular gyrus **Limbic**: Short insular gyri
Awareness	**Frontal**: Lateral orbital sulcus, Medial orbital sulcus, Orbital gyri, Fronto-marginal gyrus and sulcus, Suborbital sulcus, Transverse frontopolar gyri and sulci, Superior frontal gyrus **Temporal**: Paracentral lobule and sulcus, Horizontal ramus of lateral sulcus **Parietal:** Superior parietal gyrus, aMCC, Short insular gyri	None
Communication	**Frontal**: Middle frontal sulcus, Suborbital sulcus, Fronto-marginal sulci and gyri, Transverse frontopolar gyri and sulci, Superior frontal gyrus **Temporal**: Vertical ramus of lateral sulcus **Parietal:** Superior parietal lobule **Limbic**: Short insular gyri	**Frontal**: Orbital part of inferior frontal gyrus, Suborbital sulcus. **Limbic**: aMCC, Short insular gyri
Mannerism	**Frontal**: Opercular part of inferior frontal gyrus, Lateral orbital sulcus, Middle frontal sulcus, Middle frontal gyrus, Suborbital sulcus, Fronto-marginal gyrus and sulcus, Transverse frontopolar gyri and sulci, Superior frontal gyrus **Temporal**: Horizontal ramus of lateral sulcus, Lingual gyrus, Vertical ramus of lateral fissure **Parietal:** Angular gyrus, Postcentral sulcus, Superior parietal lobule, Paracentral lobule and sulcus **Occipital**: Middle occipital sulcus, Superior occipital sulcus, Cuneus, Calcarine sulcus **Limbic**: aMCC, Superior circular sulcus of insula, Short insular gyri	**Frontal**: Opercular part of inferior frontal gyrus, Suborbital sulcus **Parietal:** Paracentral lobule and sulcus, Subparietal sulcus **Occipital**: Lingual part of the medial occipito-temporal gyrus **Limbic**: aMCC, Marginal cingulate sulcus
Motivation	**Frontal**: Suborbital sulcus, Fronto-marginal gyrus and sulcus, Superior frontal gyrus, Transverse frontopolar gyri and sulci **Parietal:** Superior parietal lobule, Paracentral lobule and sulcus. **Limbic**: Short insular gyri	**Frontal**: Orbital part of inferior frontal gyrus **Limbic**: Short insular gyri

## Data Availability

Data supporting the findings of this study are either included in the manuscript and supplementary materials. The code and data are deposited at: https://github.com/siddlokray/systematic-study-of-ASD-heterogeneity-using-deep-learning
